# A Homozygous Deletion of Exon 5 of *KYNU* Resulting from a Maternal Chromosome 2 Isodisomy (UPD2) Causes Catel-Manzke-Syndrome/VCRL Syndrome

**DOI:** 10.3390/genes12060879

**Published:** 2021-06-07

**Authors:** Isabel Schüle, Urs Berger, Uta Matysiak, Gunda Ruzaike, Brigitte Stiller, Martin Pohl, Ute Spiekerkoetter, Ekkehart Lausch, Sarah C. Grünert, Miriam Schmidts

**Affiliations:** 1 Department of Pediatrics and Adolescent Medicine, University Hospital Freiburg, Freiburg University Faculty of Medicine, Mathildenstrasse 1, 79106 Freiburg, Germany; isabel.schuele@uniklinik-freiburg.de (I.S.); urs.berger@uniklinik-freiburg.de (U.B.); uta.matysiak@uniklinik-freiburg.de (U.M.); gunda.ruzaike@uniklinik-freiburg.de (G.R.); martin.pohl@uniklinik-freiburg.de (M.P.); ute.spiekerkoetter@uniklinik-freiburg.de (U.S.); ekkehart.lausch@uniklinik-freiburg.de (E.L.); sarah.gruenert@uniklinik-freiburg.de (S.C.G.); 2 Department of Congenital Heart Disease and Pediatric Cardiology, Faculty of Medicine, University of Freiburg, 79106 Freiburg, Germany; brigitte.stiller@universitaets-herzzentrum.de; 3 Genome Research Division, Human Genetics Department, Radboud University Medical Center and Radboud Institute for Molecular Life Sciences (RIMLS), Geert Grooteplein Zuid 10, 6525 KL Nijmegen, The Netherlands

**Keywords:** VCRL, Catel–Manzke, *KYNU*, CAKUT, renal hypodysplasia, hypoplastic left heart, hyperphalangism

## Abstract

Vertebral, Cardiac, Renal and Limb Defect Syndrome (VCRL), is a very rare congenital malformation syndrome. Pathogenic variants in *HAAO* (3-Hydroxyanthranilate 3,4-dioxygenase), *NADSYN1* (NAD+ Synthetase-1) and *KYNU* (Kynureninase) have been identified in a handful of affected individuals. All three genes encode for enzymes essential for the NAD+ de novo synthesis pathway. Using Trio-Exome analysis and CGH array analysis in combination with long range PCR, we have identified a novel homozygous copy number variant (CNV) encompassing exon 5 of *KYNU* in an individual presenting with overlapping features of VCRL and Catel–Manzke Syndrome. Interestingly, only the mother, not the father carried the small deletion in a heterozygous state. High-resolution SNP array analysis subsequently delineated a maternal isodisomy of chromosome 2 (UPD2). Increased xanthurenic acid excretion in the urine confirmed the genetic diagnosis. Our findings confirm the clinical, genetic and metabolic phenotype of VCRL1, adding a novel functionally tested disease allele. We also describe the first patient with NAD+ deficiency disorder resulting from a UPD. Furthermore, we provide a comprehensive review of the current literature covering the genetic basis and pathomechanisms for VCRL and Catel–Manzke Syndrome, including possible phenotype/genotype correlations as well as genetic causes of hypoplastic left heart syndrome.

## 1. Introduction

Vertebral, Cardiac, Renal and Limb Defect Syndrome (VCRL) is a rare autosomal-recessively inherited condition associated with cardiac malformations, including hypoplastic left heart syndrome, short stature, dysmorphic facial features such as microcephaly, low set ears and a flat nasal bridge, skeletal malformations as vertebral segmentation defects, rhizomelic shortening of the limbs, finger hyperphalangism and kidney malformations. Possible additional features include sensorineural hearing loss and developmental delay [1,2,3]. The underlying genetic causes were first elucidated by Shi et al., 2017 [1]. The authors described pathogenic variants in the *KYNU* and *HAAO* gene to be causative for this malformation syndrome (VCRL1, OMIM **#** 617660 and VCRL2, OMIM **#** 617661). Recently, Szot et al. identified pathogenic variants in *NADSYN1* and hereby the third causative gene for the syndrome (VCRL3, OMIM **#** 618845) [2]. All three enzymes play a role in the biosynthesis of nicotinamide adenine dinucleotide (NAD+) cofactors from tryptophan and pathogenic mutations in *HAAO* as well as *KYNU* were found to cause reduced NAD+ levels in affected individuals, potentially resulting in the distinct malformations observed in VCRL [1], however the precise underlying pathomechanism has remained elusive. Consequently, VCRL1–3 are also referred to as NAD+ deficiency related disorders [1,2]. To date, only 12 patients from 11 families with VCRL have been described in the literature (2 cases with VCRL1 and 5 cases with VCRL2 and VCRL3, respectively). All but two cases were found to suffer from congenital heart defects (two cases could not be assessed due to termination of pregnancy), including 5 individuals with hypoplastic left heart syndrome (HLHS) [1,2,3].

The phenotype of patients with VCRL is overlapping with that of CATMANS (Catel–Manzke Syndrome, CMS) patients which is caused by mutations in the *TGDS* gene that encodes for TDP-glucose 4,6-dehydratase. CMS is clinically characterized by skeletal anomalies, namely bilateral hyperphalangy causing ulnar clinodactyly, radial deviation and shortening of the index finger as well as Pierre Robin sequence which can include a cleft palate [4]. Additional manifestations include congenital heart defects, pectus deformities, joint hypermobility and hernia and in some cases development delay [5,6,7,8,9,10].

Here, we describe the identification of a novel homozygous CNV resulting from a maternal chromosome 2 isodisomy in the *KYNU* gene causing a phenotype overlapping VCRL and CMS. We further provide a review of the current literature.

## 2. Materials and Methods

### 2.1. Whole Exome Sequencing

Written consent of the probands/legal guardians was obtained and Whole Exome Sequencing (WES) for the patient and the unaffected parents was performed using the Agilent Sure Select Human whole exome kit (Agilent Technologies, Santa Clara, CA, USA) for enrichment. Sequencing was undertaken on a Nextseq 2000 machine (Illumina, San Diego, CA, USA). Read alignment and variant calling were performed with GATK (genome analysis toolkit) using default parameters with the human genome assembly hg19 (GRCh37) as reference. Variant filtering was performed focusing on protein coding changes and splice site regions based on a minor allele frequency of <1% in public databases, including the Exome Aggregation Consortium, Genome Aggregation Database, dbSNP, the 1000 Genomes Project and gnomAD. CNV calling was performed using SeqPilot software (JSI medical systems, Ettenheim, Germany).

### 2.2. CGH Array

CGH array was performed using Agilent Sure Print G3 CGH Mircoarry 4 × 180 k kit according to the manufacturers protocol (Agilent, USA).

### 2.3. SNP Array

SNP array was performed using Sure Print G3 CGH Micorarry + SNP 4 × 180 k kit according to the manufacturers protocol (Agilent, Santa Clara, CA 95051, USA).

### 2.4. Long Range Polymerase Chain Reactions (LRPCR)

Long range polymerase chain reactions was performed using the Qiagen LongRange PCR kit (Qiagen, Germantown, MD 20874, USA) using 100 ng of genomic DNA. PCR was performed as suggested by the manufacturer (annealing temperature of 62 °C, extension time of 1 min/kb 35 cycles). Gel analysis was performed using 2.5 V/cm for 5 h and 0.8% agarose gel. Primer sequences are available upon request.

### 2.5. Sager Sequencing

Polymerase chain reactions (PCR) were performed using 50 ng of genomic DNA using a standard touchdown PCR protocol with 34 cycles. PCR protocol and primer sequences are available upon request.

### 2.6. Urine Organic Acid Analysis

Urine samples (volume normalized to the urine creatinine concentration) were spiked with internal standard, acidified and organic acids were extracted with ethyl acetate. The extracts were concentrated and the organic acids were derivatized using diazomethane. Di- and tri-methylated derivatives of xanthurenic acid were formed. Methylated residues were dissolved in methanol and analyzed on a 7890 A gas chromatograph coupled to a 5975 C mass spectrometer (both Agilent Technologies, Santa Clara, CA 95051, USA). Separation was performed on a CP-Wax 58 column (25 m × 0.25 mm × 0.2 µm, Agilent, Santa Clara, CA 95051, USA) over 65 min using a temperature gradient from 50 to 260 °C. The mass spectrometer was operated in electron ionization and full scan mode, acquiring spectra in the range *m*/*z* 33–400.

## 3. Results

### 3.1. Clinical Description

**Figure 1:**
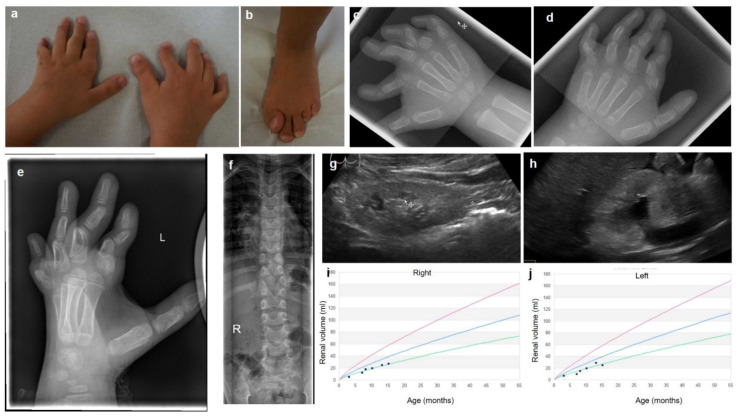
Phenotypic features resulting from KYNU loss of function. Characteristic hand and foot malformations (**a**–**e**), including hyperphalangism (**c**–**e**), lumbosacral wedge vertebrae (**f**) and bilateral renal hypodysplasia (ultrasound images, **g**,**h**; renal volumes depicted in **i**,**j**).

The affected individual has been treated in our institution since birth. Phenotypic hallmarks are shown in Figure 1. The girl was the second child of healthy non-consanguineous parents and was born at term, (39+4/7 weeks of gestation). Birth parameters were 46 cm (2 cm < P3), 3.0 kg (P10–25), head circumference 33.5 cm (P10). Shortened long bones, hypoplastic left heart syndrome (HLHS) with mitral atresia and aortic atresia, rocker-bottom feet and crossed fingers were noted during the pregnancy. HLHS was diagnosed by ultrasound shortly after birth. Norwood I surgery with a Sano Shunt was realized in the second week of life. Subsequent upper cavopulmonary connection (Norwood II) followed at the age of 5 months and implementation of a total cavopulmonary connection (TCPC) was performed at the age of 4 years without major complications. Additional balloon angioplasty was performed twice subsequently due to postoperative aortic coarcation. At the age of 5 years, she was an active child without clinical complaints. Echocardiography showed good function of the systemic right ventricle with only mild tricuspid regurgitation, on medication with enalapril and phenprocoumon.

Postnatal ultrasound examination also revealed bilateral hypoplastic kidneys. At the age of two months, the right kidney measured 3.4 cm (<P3) and the left kidney was 3.0 cm (<P3). Blood creatinine was 0.40 mg/dL, indicative of a mild impairment of renal function. At the age of one year, a mild pathological increase of kidney echogenicity was observed on ultrasound examination. At the age of 5 years, kidney sizes and volumes remained significantly below the average: the right kidney length was 5.4 cm (<P3) and the volume 27 mL (<P3), the left kidney length was 5.0 cm (P3) and the volume 25 mL (<P3), Figure 1g–j. Kidney function was slightly impaired with a blood creatinine concentration of 0.48 mg/dl (GFR 79,16 mL/1.73 m^2^, Schwartz formula, CKD stage 2) and without signs of proteinuria or hematuria.

Additionally, multiple skeletal anomalies were identified, including bilateral clinodactyly of digit IV and V, a radial deviation and membranous polydactyly on the right hand with doubling of the proximal phalanges of the right digitus II, IV and V. The feet showed a bilateral shortening of the metatarsalgia IV and hallux valgus (Figure 1a–e). Further, shortening of both humeri was noted. Radiography showed lumbosacral wedge vertebrae (Figure 1f) and a hip maturation delay was diagnosed. There were no special features of the skull or the thorax. At the age of 5.5 years, her length was 92 cm (13 cm < P3) and her weight was 15.7 kg (P3). The patient attended kindergarden and showed a favorable psychomotor development.

In summary, the patient presented with an overlapping phenotype of VCRL and Catel–Manzke Syndrome.

### 3.2. Genetic Findings

**Figure 2:**
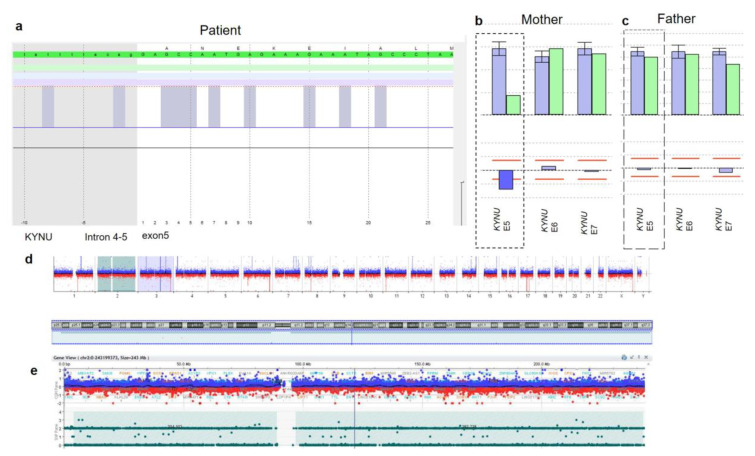
Genetic findings. Visual inspection of coverage of *KYNU* revealed absent NGS reads for exon 5 (**a**). CNV analysis of NGS data suggested a heterozygous deletion of *KYNU* exon 5 in the mother (**b**) but not the father (**c**), patient shown in green, controls in purple, analysis using SeqPilot software). SNP-chip analysis revealed loss of heterozygosity for chromosome 2 but not for other chromosomes ((**d**), upper panel). Close-up of chromosome 2 confirming loss of heterozygosity ((**e**), lower panel).

The karyotype was found to be normal and a standard Next Generation Sequencing (NGS) gene panel analysis for ciliopathy causing genes (Bioscentia, Ingelheim, Germany) did not reveal any causative variants. Likewise, no causative deletions or duplication were detected by CGH array analysis. We therefore proceeded to perform Trio-Exome analysis of the patient and both parents, including CNV analysis. Trio-Exome analysis again did not reveal any pathogenic SNVs or indels fitting with the phenotype of the patient. In particular, there were no pathogenic variants in the *TGDS* gene, which is known to be causative for CMS [6,7,8,10] nor any of the three VCRL genes. However, manual inspection of coverage of genes in which mutations have been reported to cause HLHS revealed a homozygous deletion of exon 5 of the *KYNU* gene (NM_00119924.1) in the patient (Figure 2a). Exon 5 contains 62 bp and the deletion is predicted to result in a frameshift and truncated protein (c.374_ 436del, p. 125_145delL146Yfs*15).

SeqPilot Software detected the CNV in a heterozygous state in the mother (Figure 2b) but unexpectedly not in the father (Figure 2c). To exclude a previously published larger deletion affecting *KYNU* in the father, we performed Sanger sequencing of the corresponding area of the gene [3] confirming wildtype genotypes). To exclude non-paternity, we proceeded to manually compare chosen paternal rare variants (MAF < 1%) in the patient’s exome data: while no rare paternal SNPs were identified on chromosome 2 in the patient, rare paternal SNPs on other chromosomes were present. This was confirmed by SNP array analysis, excluding non-paternity and instead showing maternal uniparenteral isodisomy of chromosome 2 (Figure 2d).

The small *KYNU* CNV detected homozygously in the patient and heterozygously in the mother was missed by the previously performed CGH array as no CGH probe located to the deleted exon. However, a probe at the intronic position chr2:143,705,606 preceeding the deleted exon was still visible, allowing us to predict the first breakpoint to lie within an approximately 8000 bp measuring DNA stretch between this intronic probe and exon 6 and the second breakpoint in the small intron 6/7. Long range PCR revealed a slightly shorter product in the patient compared to the father as well as a healthy unrelated control (Supplementary Figure S1a) and we were able to locate the breakpoints to GRCh38 chr.2:142,954,376 and chr.2:142,955,239, confirming a very small, only 863 bp measuring homozygous deletion in the patient (the proximal breakpoint is shown in Supplementary Figure S1b). This deletion was confirmed in a heterozygous state in the mother but not the father using Sanger sequencing.

### 3.3. Metabolic Findings

**Figure 3:**
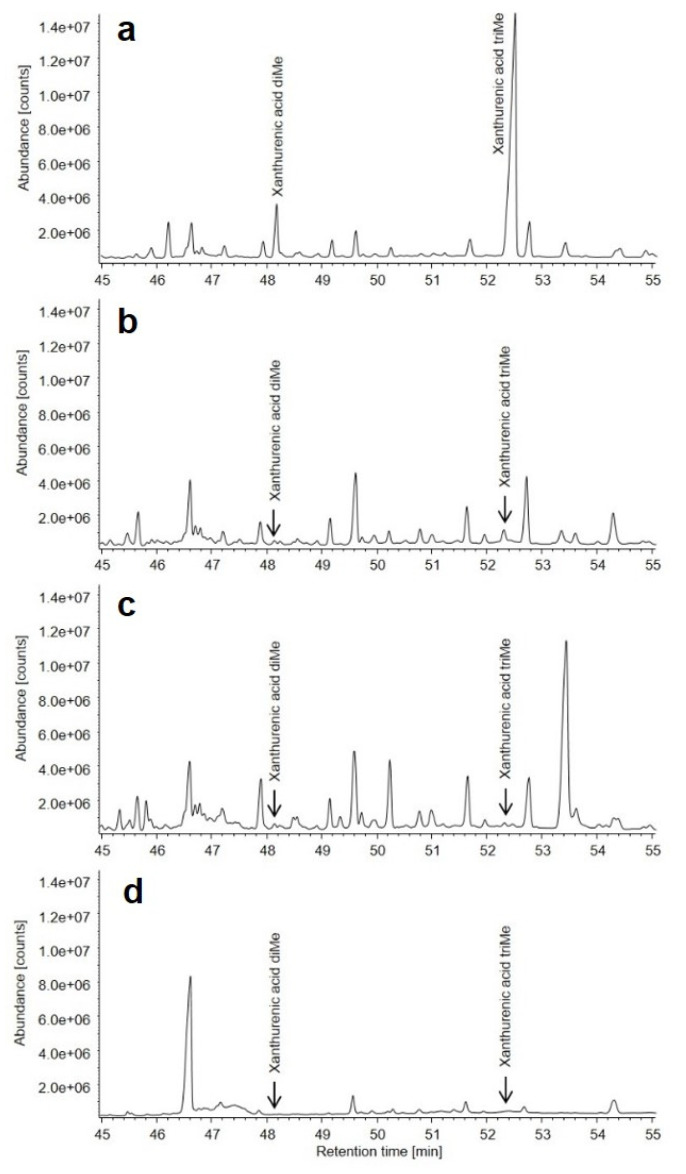
Total ion current chromatogram (*m*/*z* 33–400) of the methylated organic acid extract from urine. (**a**) Urine analysis of the affected child, (**b**) mother, (**c**) father and (**d**) a healthy age and sex matched control. The signal at 46.5 min is caffeine. The signal at 52.4 min representing tri-methylated xanthurenic acid is strongly enhanced in the sample from the affected child (**a**) while a subtle enhancement is also detected in the sample from the mother who carries the CNV in a heterozygous fashion (**b**).

We next proceeded to biochemically confirm our genetic findings, suggesting that loss of KYNU function will result in an elevated excretion of xanthurenic acid in the urine: KYNU is essential to transform 3-hydroxykynurenin (3-HK) to 3-hydroxanthranilic acid (3-HAA). KYNU dysfunctions has been previously shown to result in elevated urine xanthurenic acid levels [3]. Spot urine samples of all 3 family members and an unrelated healthy control were analyzed by gas chromatography followed by mass spectrometry (GC/MS). As expected, we detected markedly increased xanthurenic acid concentrations in the child and slightly increased levels in the mother compared to the father and the control sample (Figure 3 and Supplementary Figure S2).

## 4. Discussion

Here, we have identified a novel CNV in *KYNU* causing VCRL with homozygosity in the patient resulting from a maternal isodisomy of chromosome 2 (UPD2). Reports in the literature show that a unipaternal iso- or heterodisomy of chromosome 2 does not lead to phenotypic anomalies of affected individuals, suggesting that there are no imprinted genes located on chromosome 2 [11,12,13,14].

Identification of maternal UPD2 causing homozygosity of the *KYNU* exon 5 CNV is important for genetic counselling of our family as in this case, the recurrence risk in the family is considerably lower in comparison to a recessively inherited disorder.

**Figure 4:**
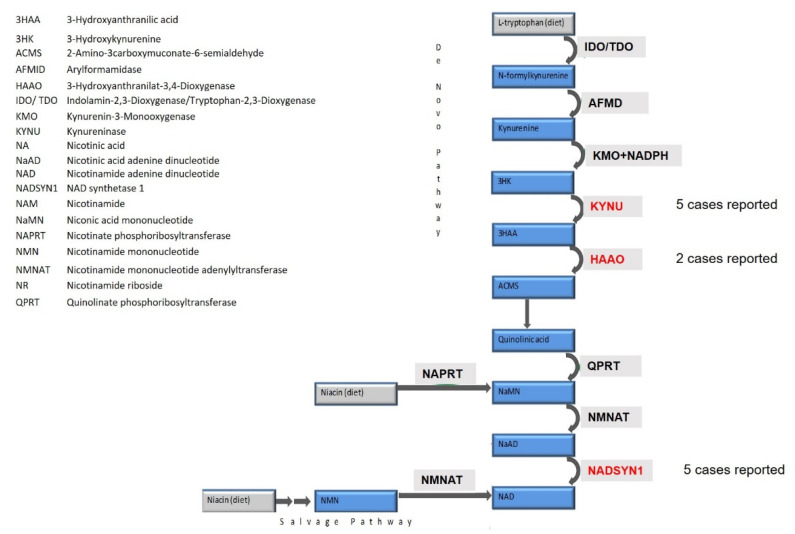
Schematic diagram of NAD+ biosynthesis via the de novo pathway and the salvage pathway. The de novo biosynthesis starts from dietary L-tryptophan which is enzymatically converted in a series of reactions to quinolinic acid. These reactions include cleavage of 3HK to 3HAA catalyzed by KYNU and oxidation of 3HAA to ACMS catalyzed by HAAO. QPRT subsequently converts quinolinic acid to NaMN which in turn is then converted to NaAD by NMNAT. NADSYN1 catalyzes the final step of the de novo pathway: amidation of NaAD to NAD. The salvage pathway starts from dietary uptake of several niacin equivalents: NA, NAM or NR. NA is converted to NaMN by NAPRT and then converted to NAD by NMNAT and NADSYN1. Both NAM and NR are converted to NMN and then converted to NAD by NMNAT.

*KYNU* encodes the enzyme kynureninase, which plays a role in the tryptophan catabolic pathway and is needed for the biosynthesis of NAD+. NAD+ is either produced by the “NAD+ de novo synthesis pathway” from dietary tryptophan, requiring among other enzymes KYNU, HAAO and NADSYN1 or by the “NAD+ salvage pathway” from dietary niacin independent from those enzymes. Disturbances of the de novo pathway by pathogenic mutations in critical enzymes can cause VCRL (Figure 4).

Loss of function mutations in *KYNU* lead to an accumulation of the upstream metabolites and elevated levels of 3-hydroxykynurenine and xanthurenic acid can be detected in the urine and plasma of affected individuals. Likewise, downstream metabolites, namely picolinate and quinolinate in urine and NAD+ in plasma, are found to be reduced in patients [1,3]. NAD+ deficiency during embryonic development rather than accumulation of metabolites upstream the metabolic block has been proposed as a critical factor for the pathogenesis of VCRL [1]. NAD+ acts as an essential coenzyme in hundreds of redox reactions and is important for protein–protein interactions, epigenetics, mitochondrial function, DNA repair, cell division, immune response and inflammation. This variety of functions explains the multiorgan involvement associated with *KYNU* loss of function [15,16].

Interestingly, the heart and the kidneys, which are usually severely affected in patients with VCRL, are among the organs with the highest NAD+ levels, the highest numbers of mitochondria and are among the greatest oxygen consumers of all organs in the body [17].

Chronic as well as acute kidney and heart stresses have been associated with decreased NAD levels [18,19]. Two classes of NAD+ consuming enzymes highlight the importance of NAD+ for kidney and heart function: (1) Sirtuins that regulate mitochondrial processes including the resilience to oxidative stress and cell survival and (2) ADP-ribosyltransferases and the ADPR cyclases, which play a role in DNA damage repair and calcium signaling pathways [20,21].

Christensen et al. described two brothers with a missense variant in *KYNU*, resulting in a threonine to alanine amoinoacid exchange (p.Thr198Ala), presenting with hydroxykynureninuria and xanthurenic aciduria but without congenital malformations [22]. A residual activity of kynureninase in these patients with consequently higher levels of plasma NAD+ could explain the distinct phenotype, presuming that only the decrease of NAD+ level below a certain threshold during critical timepoints of organogenesis leads to congenital malformations [1].

**Figure 5:**
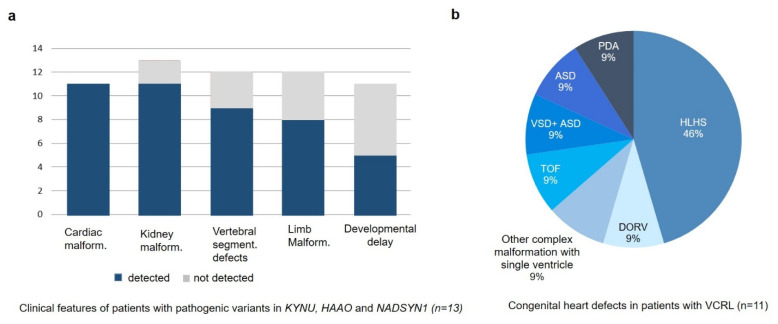
Clinical features of VCRL cases reported to date. (**a**) Main clinical features of patients described in literature with pathogenic variants in *KYNU*, *HAAO* and *NADSYN1* (*n* = 13). (**b**) Cardiac phenotype of patients with pathogenic variants in *KYNU*, *HAAO* and *NADSYN1* (*n* = 11). *ASD*, Atrial septal defect; *DORV*, Double outlet right ventricle; *HLHS*, Hypoplastic left heart syndrome; *PDA*, Patent ductus arteriosus; *TOF*, Tetralogy of fallot; *VSD*, Ventricular septal defect.

Why some of the patients with NAD+ deficiency syndrome show a more severe cardiac phenotype in comparison to others is speculative. Presuming that the amount of NAD+ in the embryonic development is an essential factor, it is imaginable that patients with less severe malformations have either a higher residual enzyme activity or have higher NAD+ level due to environmental factors such as higher nutritional niacin intake of the mother during pregnancy [1]. On the other hand, reduced maternal NAD+ during pregnancy due to health factors such as diabetes could result in more severe phenotypes in the child [23]. Due to the small number of cases reported in the literature, phenotype-genotype correlations in VCRL have to be considered with caution, however the available data do not suggest a more severe phenotype in cases with presumable null alleles versus presumable hypomorphic missense alleles (Table 1). A summary of clinical features of genetically confirmed VCRL patients from the literature is shown in Table 1 and Table 2 and Figure 5a.

Our patient was clinically diagnosed with Catel–Manzke Syndome (CMS)/Manzke Dysostosis, a phenotype overlapping with VCRL. Genetically, the identified CNV in *KYNU* suggests VCRL as diagnosis. Bilateral hyperphalangy with an accessory bone inserted between the second metacarpal and the phalanx, resulting in radial deviation of the index finger, has been described as a cardinal feature for CMS, however only nine out of twelve reported patients with *TGDS* mutations show this phenotype and also four out of twelve VCRL patients with pathological variants in *KYNU*, *HAAO* or *NADYSN*1 show this malformation. All reported CMS patients with *TGDS* mutations were diagnosed with Pierre Robin sequence while none of the patients with VCRL were. On the other hand, (mild) developmental delay more often occurs in patients with VCRL. Especially the cardiac and renal phenotype of patients with VCRL seems to be more severe in comparison to those with CMS. All screened patients with VCRL were diagnosed with congenital heart defect, including five patients with HLHS, one patient with double outlet right ventricle (DORV), one patient with tetralogy of fallot (TOF), one patient with combined atrial septal defect (ASD) and ventricular septal defect (VSD), one patient with isolated ASD and one patient with patent ductus arteriosus. Observed cardiac malformations of patients with VCRL are shown in Figure 5b.

Interestingly, HLHS is the most frequent congenital heart defect observed in VCRL. HLHS is a rare condition with a prevalence of 1.6 per 10,000 live births [24]. The exact developmental mechanisms of the syndrome are not understood and only few monogenetic causes have been identified to date, often causing syndromal disease patterns (Supplementary Table S1) [25,26]. However, a 500-fold increased risk for individuals with a sibling suffering from a congenital heart defect [27] strongly suggests further contributing genetic factors.

Future research is needed to determine the exact NAD+ dependent processes responsible for the disruption of certain stages of organogenesis especially affecting heart, kidneys and skeleton in VCRL patients. Shi et al. showed a rescue of the clinical phenotype in *KYNU*−/− mice embryos by dietary supplementation of niacin of the pregnant mice [1]. It is possible that niacin supplementation during pregnancy could also minimize the risk of recurrence in affected families with NAD deficiency disorders, but this is to date speculative.

## 5. Conclusions

In summary, we describe a case of NAD+ deficiency syndrome due to a homozygous CNV on the basis of a maternal UPD2. Our findings confirm that patients with NAD+ deficiency syndromes can display characteristics previously described for CMS and therefore mutations in enzymes of the tryptophan catabolic pathway should be excluded in patients diagnosed with CMS not carrying mutations in *TGDS*.

## Figures and Tables

**Table 1:** Genetic, clinical and metabolic phenotype of patients with VCRL.

Literature	Gene	Genotype	Cardiac Phenotype	Renal Phenotype	Skeletal Phenotype	Neurological Features	Additional Features	Metabolic Findings	NAD+ Level
Patient reported in this report	KYNU	863 bp deletion (g.del142954376-g.142955239): loss of exon 5 Hom	HLHS	Bilateral hypoplasia	DVS clinodactily of 4th and 5th fingers, right-sided polydactily, feet: Shortening of the metatarsalia IV, hallux valgus, shortening of the upper arms	Normal develop-ment	Sacral hyperpegmentation	Increased Xanthenu-renic acid excretion in the urine	n.a.
Shi et al., 2017	KYNU	c.170-1G>T (p.V57Efs*21) Hom	PDA	Bilateral hypoplasia	DVS, Talipes, syndactyly, rhizomelia	-	Low-set ears, anterior anus	n.a.	n.a.
Shi et al., 2017	KYNU	c.468T > A (p.Y156*) het + c.1045_1051delTTTAAGC (p.F349Kfs*4) het	HLHS	Solitary kidney, CKD	DVS, Short Stature, bilateral shortening of humeri and femora	Speech delay	-	* 3HK, 161 times the mean	* NAD(H), 1/7th of the mean
Ehmke et al., 2019	KYNU	delEx1-8 het + c.1282C > T; (p.Arg428Trp) het	HLHS	-	DVS, MD	Mild DD	Hepato-megaly, microretro-gnathia, facial dysmor-phism	** 3HK, 56 times the mean	
Ehmke et al., 2019	KYNU	c.989G > A (p.Arg330Gln) Hom	Tetralogy of Fallot, ALCAPA	-	DVS, MD, long thumbs	DD, muscular hypotonia, microcephaly	Senso-neuronal hearing loss, facial dysmor-phism	** 3HK, 45 times the mean	
Ehmke et al., 2019	KYNU	c.326G > C (p.Trp109Ser) Hom	Secundum ASD, subaortic VSD	Unilateral renal agenesis	Brachydac-tyly, Clinodactyly, 2nd/3rd-toe syndactyly, MD	Mild DD, microcephaly	Bilateral single transverse palmar crease, joint hypermo-bility, facial dysmor-phism	n.a	n.a
Shi et al., 2017	HAAO	c.483dupT (p.D162*) Hom	ASD	Hypoplasiavesicoure-teral reflux	DVS Short stature, talipes	DD	Sensorineural hearing loss, laryngo-malacia	* 3HAA, 64 times the mean	* NAD+, 1/3rd of the mean
Shi et al., 2017	HAAO	c.558G > A (p.W186*) Hom	HLHS	Hypodys- plasia	DVS -	Palsy of left vocal cord	Sensori-neural hearing loss on left side	* 3HAA, 385 times the mean	* NAD(H), 1/4th of the mean
Szot et al., 2020	NADSYN1	c.1717G > A (p.Ala573Thr) Hom	Borderline HLHS hypoplastic mitral valve, small bicuspid aortic valve, hyperplasia/coarctation of the aortic arch ALCAPA	Absent left kidney	Thoracic vertebral defect, bilateral shortening of humeri and femora	-	Sacral dimple	n.a.	n.a.
Szot et al., 2020	NADSYN1	c.1717G > A (p.Ala573Thr) Hom	Absent left ventricle and pulmonary trunk, right ventricular outlet to the aorta	Bilateral hypoplastic kidneys	DVS, bilateral shortening of humeri and femora	-	-	n.a.	n.a.
Szot et al., 2020	NADSYN1	c.1717G > A (p.Ala573Thr) c.1819del (p.Val607Trpfs*30)	DORV, TGA in side by side orientation, VSD, PDA, left aortic arch	Mild hyperecho-genic renal cortex	DVS, scoliosis, rib abnormalities, bilateral shortening of humeri and femora, bowing of lower extremities	n.a.	n.a.	n.a.	n.a
Szot et al., 2020	NADSYN1	c.145T > C (p.Cys49Arg) c.395G > T (p.Trp132Leu)	n.a. termination of pregnancy at 16 w	Oligohy-dramnion, bilateral renal agenesis	n.a.	n.a.	n.a.	n.a.	n.a
Szot et al., 2020	NADSYN1	c.735T > A (p.Cys245*) c.1839C > G (p.Tyr613*)	n.a. termination of pregnancy at 16 w	Left renal and ureter agenesis, edema	Small thorax, microme-lia, bilateral club feet	Hydrocephalus	Facial dysmor-phism echogenic bowel, polysplenia, pulmonary hypoplasia	n.a.	n.a

* Level in proband plasma vs. mean in unaffected family members. ** Level in proband plasma vs. mean in healthy control individuals. HLHS (Hypoplastic left heart), DORV (Double outlet right ventricle, TGA (Transposition of the Great Arteries), VSD (ventricular septal defect), PDA (patent ductus arteriosus), ASD (Atrial septal defect), ALCAPA (Anomalous left coronary artery from the pulmonary artery), DVS (Defects in vertebral segmentation), MD (Hyperphalangism/Manzke Dysostos, DD (Development delay).n.a. (not available).

**Table 2:** Phenotype of patients with Catel–Manzke Syndrome and bilateral pathogenic variants in TGDS.

Literature	Number of Patients	Cardiac Malformations	Kidney Malformations	Spine/Thorax	Manzke Dysostosis	Shortening of the Limbs	Clinodactily/Brachydactily	Pierre Robin Sequence	Neurological Features	Additional Features
Ehmke et al., 2014	7	2/7 (2VSD)	0/7	1/7 (pectus deformity)	7/7	1/7	5/7	7/7	0/7	Hearing loss
Pferdehirt et al., 2015	1	1/1 (PDA)	0/1	1/1 (pectus deformity)	1/1	0/1	1/1	1/1	0/1	Laryng-omalacia
Schoner et al., 2017	1	1/1 (VSD, coarcation of aorta)	0/1	0/1	1/1	1/1	1/1	1/1	n.a.	n.a
Miller et al., 2019	1	1/1 (ASD, VSD)	0/1	1/1 (scoliosis)	0/1	0/1	0/1	1/1	0/1	n.a.
Boschann et al., 2020	2	0/2	0/2	1/2 (pectus deformity	0/2	2/2	2/2	2/2	0/1	Hip dysplasia
total	12	5/12	0/12	4/12	9/12	4/12	9/12	12/12	0/11	3/10

n.a. (not available).

## Data Availability

Data and materials are available on personal request.

## Supplementary Materials

The following are available online at https://www.mdpi.com/article/10.3390/genes12060879/s1, Table S1: Known genetic causes of HLHS; Figure S1: Breakpoint analysis using Long range PCR and Sanger sequencing; Figure S2: Gas chromatography and mass spectrometry standards.
