# Supplementary material for: A Homozygous Deletion of Exon 5 of KYNU Resulting from a Maternal Chromosome 2 Isodisomy (UPD2) Causes Catel-Manzke-Syndrome/VCRL Syndrome
Source: Genes (Basel). 2021 Jun 7;12(6):879. doi: 10.3390/genes12060879 (PMC8227568; doi:10.3390/genes12060879)
Supplement: Supplementary file 1 [file genes-12-00879-s001.zip › genes-1228798-supplementary.pdf]

Suppelementary Table S1 Known genetic causes of HLHS			
**Gene**	**Genomic location**	**Function**	**Literature**
**HLHS without extracardiac involvement**			
**NKX2-5**	5q35.1	Encodes for a homeobox-containing transcription factor important for tissue differentiation and development of the heart	Stallmeyer et al., 2010 (4)
**HAND1**	5q33.2	Encodes for a basic helix-loop-helixtranscription factor that is essential for mammalian heart development	Reamon-Buettner et al., 2008 (5).
**rbFOX2**	22q12.3	Encodes for a regulatory protein importatnt for tissue-specific exon splicing in pre-mRNAs	Homsy et al., 2015 (6)
**ERBB4**	2q34	Encodes a tyrosine protein kinase; required for muscle differentiation cardiomyocyte proliferation	McBride et al., 2011 (7)
**GJA1**	6q22.31	Encodes for a protein that is a component of gap junctions in the heart	Dasgupta et al., 2001 (8)
**MYH6**	14q11.2	Encodes for a protein involved in myocyte contractility	Theis et al., 2015 (9)
**TAB2**	6q25.1	Encodes for a protein that play a role in the development of the cardiovascular System	Cheng et al., 2020 (10)
**HLHS with additional extracardiac features**			
**NOTCH1;** Aortic valve disease 1 Adams-Oliver syndrome 5	9q34.3	Encodes the Notch 1 protein receptor, important for NOTCH signalling pathway	Iascone et al., 2012, (11)
**ETS1;** Jacobsen Syndrome	11q24.3	Encodes for a transcription factor important for regulation of expression of genes controlling endothelial cell migration and invasion	Glessner et al., 2014 (12)
**TBX5;** CHM, some cases with extracardiac manifestations	12q24.1	Encodes for a protein that is important for growth and development of the interventricular septum of the heart	Takeuchi et al, 2003 (13)
**FOXC2;** Lymphedema-distichiasis syndrome	16q24.1	Encodes a transcription factor involved in the development of the cardiovascular system	Stankiewicz et al, 2009(14)
**HAAO;** VCRL2	2p21	Encodes for an enzyme involved in the de novo NAD(H) synthesis pathway	Shi et al., 2017 (1)
**KYNU;** VCRL1	2q22.2	Encodes for an enzyme involved in the de novo NAD(H) synthesis pathway	Shi et al., 2017 (1)
**NADSYN;** VCRL3	11q13.4	Encodes for an enzyme involved in the de novo NAD(H) synthesis pathway	Szot et al., 2020 (2)
**Chromosomal aberrations associated with HLHS**			
**Monosomy X,** Turner Syndrome			Natowicz et al, 1988 (15)<br>Mazzanti et al, 1998 (16)<br>Loscalzo et al., 2005 (17)
**Trisomy 18;** Edward Syndrome			Natowicz et al, 1988 (15)<br>Ferencz et al., 1997 (18)
**Trisomy 13;** Patau Syndrome			Natowicz et al, 1988 (15)<br>Ferencz et al., 1997 (18)
**Terminal deletion 11q;** Jacobsen Syndrome			Grossfeld et al., 2004 (19)
**Terminal deletion 15q26;**			Lalani et al., 2013 (20)
**Terminal deletion 21q22.3**			Ciocca et al., 2015 (21)

Supplementary Table S1

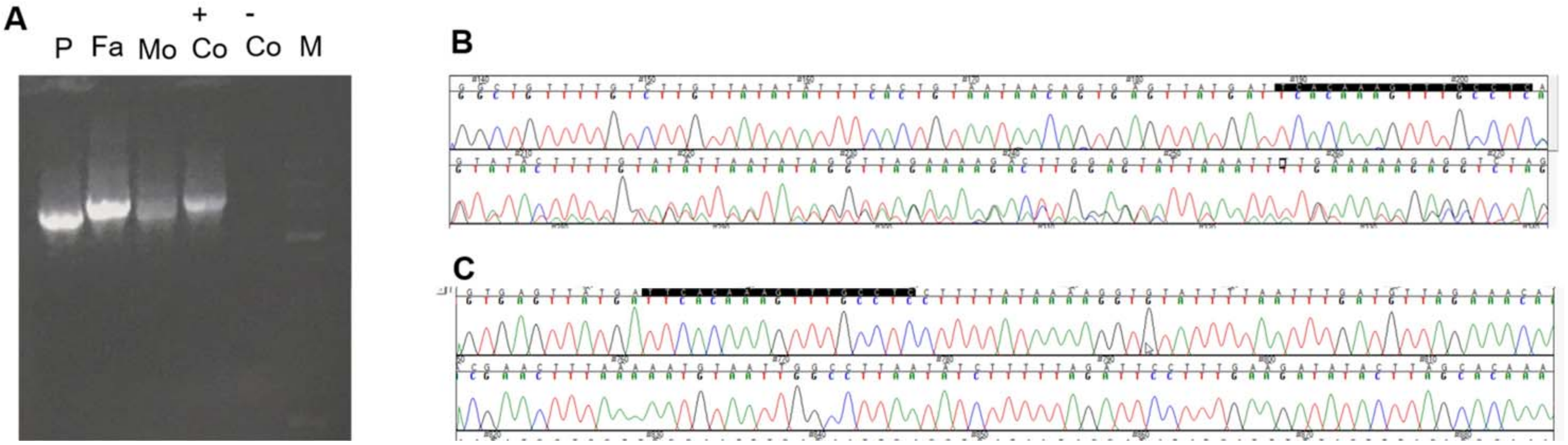

Supplementary Figure S1. Breakpoint analysis using Sanger sequencing. (A) Gel electrophoresis of long-range PCR products of the affected child (P), the father (fa), the mother (mo) positive control (control DNA, Co) and negative control (water, Co -) and marker (M) shows an approximately 1 kb smaller product in the patient as well as the mother compared to father and control. (B) Sanger analysis of the long range product shows the 5`breakpoint in the mother heterozygously (double peaks) while wildtyp sequence is detected at this position in the father (C).

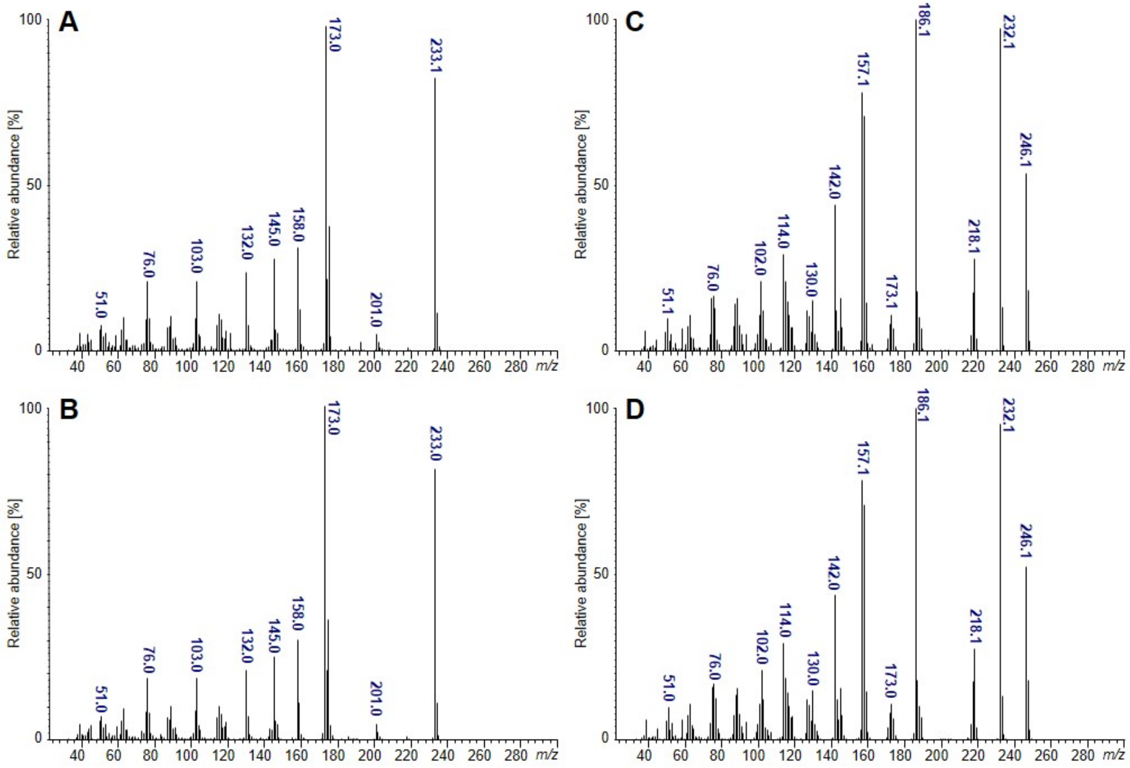

Supplementary Figure S2. Xanthurenic acid chemical reference standard. Full scan positive electron ionization mass spectra of A) signal at 48.2 min in chromatogram A (Figure 3), B) signal at 48.2 min of the methylated Xanthurenic acid chemical reference standard (presumably the di-methylated product), C) signal at 52.4 min in chromatogram A (Figure 3), and D) signal at 52.4 min of the methylated Xanthurenic acid chemical reference standard (presumably the tri-methylated product).
